# Measuring Interoception: The CARdiac Elevation Detection Task

**DOI:** 10.3389/fpsyg.2021.712896

**Published:** 2021-08-19

**Authors:** Sonia Ponzo, Davide Morelli, Chatrin Suksasilp, Massimo Cairo, David Plans

**Affiliations:** ^1^Huma Therapeutics Ltd., London, United Kingdom; ^2^Department of Engineering Science, Institute of Biomedical Engineering, University of Oxford, Oxford, United Kingdom; ^3^Department of Experimental Psychology, University of Oxford, Oxford, United Kingdom; ^4^INDEX Group, Department of Science, Innovation, Technology, and Entrepreneurship, University of Exeter, Exeter, United Kingdom

**Keywords:** interoception, mental health, cardiac perception, cardioception, psychopathology, CARED task

## Abstract

Interoception has increasingly been the focus of psychiatric research, due to its hypothesized role in mental health. Existing interoceptive tasks either suffer from important methodological limitations, impacting their validity, or are burdensome and require specialized equipment, which limits their usage in vulnerable populations. We report on the development of the CARdiac Elevation Detection (CARED) task. Participants’ heart rate is recorded by a wearable device connected to a mobile application. Notifications are sent to participants’ mobile throughout the day over a period of 4 weeks. Participants are asked to state whether their heart rate is higher than usual, rate their confidence and describe the activity they were involved in when the notification occurred. Data (*N* = 30) revealed that 1/3 of the sample was classified as interoceptive and that participants presented overall good insight into their interoceptive abilities. Given its ease of administration and accessibility, the CARED task has the potential to be a significant asset for psychiatric and developmental research.

## Introduction

The study of interoception, namely the sense of the state of one’s own body (Craig, 2002), has received increased attention in the past few years due to its links to mental and physical health (Khalsa et al., 2018). The role of interoception in disrupted physical and mental health processes is hypothesized to be so crucial that some refer to it as the P-Factor underlying psychopathology (Caspi et al., 2014; Quattrocki and Friston, 2014; Barrett and Simmons, 2015; Murphy et al., 2017; Khalsa et al., 2018).

Despite the importance of accurately measuring interoception, numerous issues exist with the most widely employed tasks, the heartbeat counting and the heartbeat detection tasks. The heartbeat counting task (HCT; Dale and Anderson, 1978; Schandry, 1981) requires participants to silently count the number of occurring heartbeats in a predefined time window. The number of heartbeats counted by the participant is then compared to the number of recorded heartbeats, thus obtaining an index of accuracy. In the HCT, however, inference about one’s resting heart rate can be used to estimate the number of heartbeats occurring in each time window (Windmann et al., 1999), thus making it hard to reliably capture interoceptive accuracy. In order to avoid potential confounds related to knowledge of one’s own heart rate, the HCT has often been paired with control tasks (e.g., participants are asked to count seconds instead of heartbeats). Nevertheless, such control tasks do not allow to exclude the possibility of participants relying on external (i.e., non-interoceptive) factors when estimating their heart rate during the HCT (see Desmedt et al., 2018 for a recent study investigating non-interoceptive processes involved in the HCT). The heartbeat detection task (HDT; Whitehead et al., 1977; Katkin et al., 1983) presents participants with a visual or auditory stimulus that is either in synchrony with their recorded heartbeat or offset by a degree (384 ms in the original paradigm). Accuracy is defined based on the number of correctly classified trials. Nevertheless, individual differences in heartbeat perception make the use of only two delays (i.e., in synchrony and out of synchrony) significantly restrictive (Clemens, 1984; Brener and Kluvitse, 1988; Brener et al., 1993; Brener and Ring, 2016).

Though psychophysics variants [e.g., the method of constant stimuli (MCS) and 6-alternative-forced-choice (6AFC) designs; Clemens, 1984; Yates et al., 1985; Brener and Kluvitse, 1988; Brener et al., 1993; Ring and Brener, 2018], as well as more recent advances in the field (the PAT task; Plans et al., 2020), tried to overcome these issues, none of these tasks is suitable for measuring interoception over time in an ecologically valid fashion. In particular, these tasks only assess interoception in one state of physiological arousal (usually rest). Given the evidence for intra-individual covariation in cardiodynamics and interoceptive accuracy (Jones and Hollandsworth, 1981; Schandry et al., 1993), a priority in interoception research is to develop a task that captures individual differences in interoception over an ecological range of cardiac activity rather than at complete rest. Moreover, remotely deployed and simple procedures for measuring interoception present a particularly valuable tool for clinical and developmental research, in which complex tasks like the MCS are more difficult to administer. Furthermore, tasks measuring interoception over time allow us to capture different dimensions of interoceptive abilities. A recent model proposed by Murphy et al. (2020) highlights the theoretical importance of distinguishing between subjective and objective interoceptive measurements as well as accuracy and attention, thus proposing that the underlying structure of interoception may be a 2 × 2 factorial one (i.e., where accuracy can be related to one’s own performance or one’s own beliefs, with the same being true for attention). The HCT, HDT, and MCS tasks described above aim to measure only interoceptive accuracy. However, none of these tasks also incorporates a measure of interoceptive attention, i.e., the extent to which interoceptive signals are the object of one’s attention (also referred to as interoceptive “sensibility”; Garfinkel et al., 2015; Khalsa et al., 2018). Measuring individuals’ abilities to accurately detect and classify interoceptive signals is undoubtedly crucial to assess potential interoceptive disruptions; however, capturing natural tendencies to pay attention to interoceptive stimuli may prove particularly useful for training paradigms aimed at improving interoceptive abilities in everyday life.

This paper presents data from a relatively small sample (*N* = 30) of healthy participants to illustrate a novel ecological interoceptive task, the CARdiac Elevation Detection (CARED) Task, aiming at capturing different components of objective interoceptive accuracy, and elements of attention, throughout a period lasting a minimum of 4 weeks. Participants wore a device collecting continuous heart rate data for the whole duration of the study. This device (a smartwatch) was connected to a mobile application that sent notifications to the participants according to a predefined algorithm. Participants were asked to state whether their heart rate was higher than usual, report their confidence in their judgments and freely describe what kind of activities they were involved in the 30 min prior to receiving the notification. Any answer related to very high intensity activities (e.g., exercise) or highly emotional states (e.g., crying or fighting with a loved one) was then discarded from subsequent analyses to minimize the confounding effect of participants potentially using knowledge about their own heart rate to make their judgment instead of basing it on perception.

Participants were then classified as either interoceptive or non-interoceptive according to how their performance compared to a distribution of reference. It was predicted that approximately 1/3 of the sample, in line with the literature (Brener and Ring, 2016), would be classified as interoceptive. Their insight into their own performance (interoceptive awareness) was assessed using confidence ratings to predict correct and incorrect responses, with the idea that higher confidence ratings would be associated with correct responses. Furthermore, potential dissociations between objective and subjective interoceptive abilities, namely subjective interoceptive accuracy and subjective interoceptive attention, were explored using well-established self-reported measures of interoception (the Interoceptive Accuracy Scale; Murphy et al., 2020, and the Body Perception Questionnaire, – awareness subscale; Porges, 1993). Finally, the relationships between mental health components (e.g., anxiety, depression, and stress) and interoceptive abilities were explored to better define potential interoceptive disruption in subclinical manifestations.

## Materials and Methods

### Participants

Fifty-two healthy participants were recruited via social media adverts. Participants were excluded if they were pregnant, or with diagnosed psychiatric or neurological disorders. Of the recruited participants, 10 became unresponsive prior to receiving the wearable device and 12 withdrew their participation. Hence, the final sample comprised 30 participants (16 females, age range: 18–51, *M* = 27.43, *SD* = 9.01). All participants signed electronic informed consent and the study was approved by a local ethics committee.

### Materials

A smartphone application was developed in React Native (TypeScript/JavaScript) for the User Interface and native Android code (Kotlin) for Bluetooth communication with the wearable device. The wearable device (Lifesense Band 2, Transtek Medical Electronics) collected heart rate data by photoplethysmography sensors (see Cropley et al., 2017; Morelli et al., 2018 for validation data), with the average heart rate over 1 min of heart activity being measured every 10 min. Finally, at baseline, participants completed a demographics questionnaire (age, gender, height, and weight) as well as the following questionnaires: the Interoceptive Accuracy Scale (IAS; Murphy et al., 2020), the Body Perception Questionnaire (BPQ – awareness subscale; Porges, 1993), the State-Trait Anxiety Inventory 6 items short form (STAI-6 SF; Marteau and Bekker, 1992), and the Depression Anxiety Stress Scale 21 items (DASS-21; Lovibond and Lovibond, 1996).

### Design and Procedure

**FIGURE 1:**
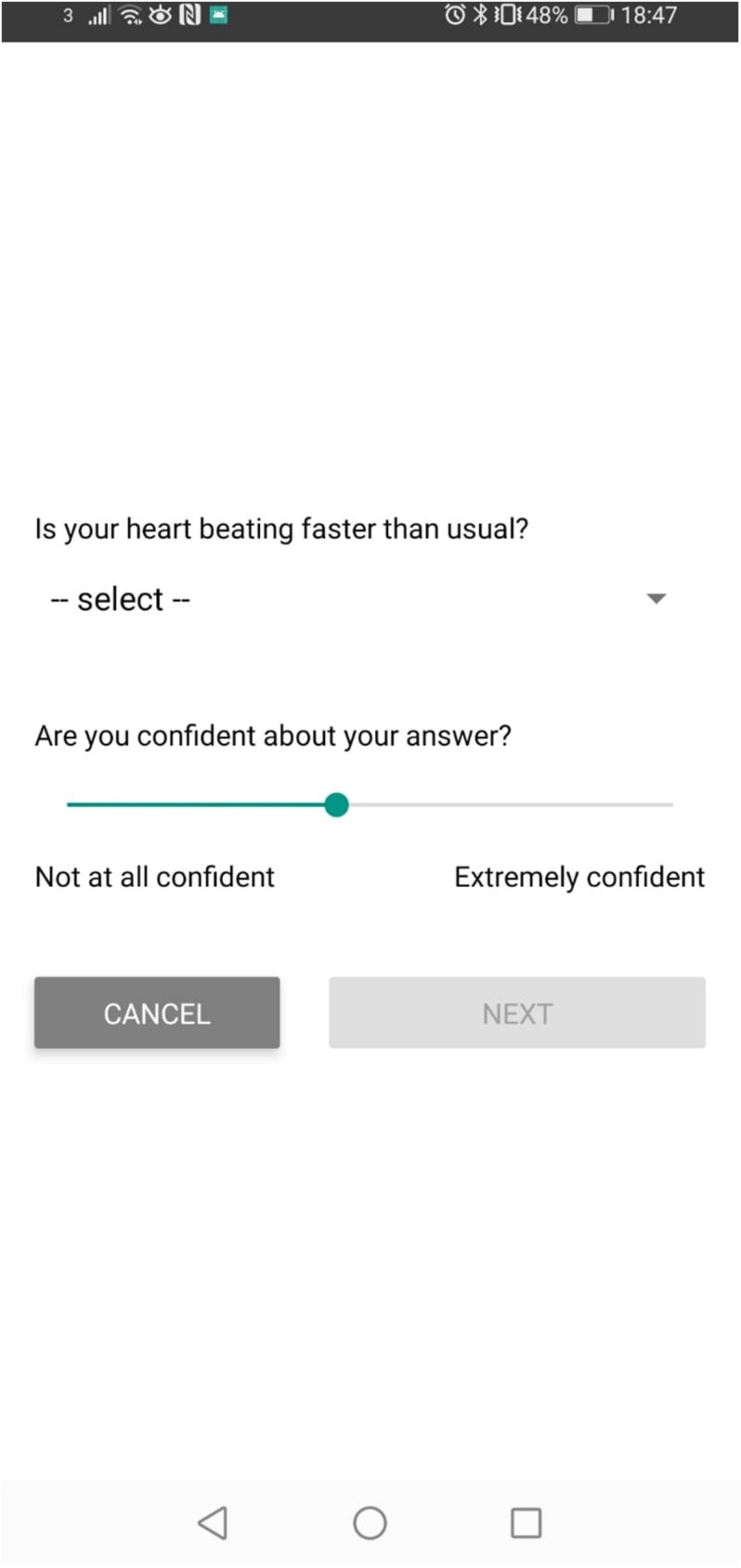
Example of a question screen comprising the heart rate judgment and the confidence rating. Following this screen, participants were presented with a free text box and asked to report what they were doing in the 30 min preceding the notification.

Participants were asked to continuously wear the wrist device for the whole duration of the study and were informed that they would be required to pay attention to the app notifications between 9 am and 9 pm. When a notification was triggered by the algorithm (the frequency of which is explained below), participants were prompted to first answer a yes/no question (i.e., “Is your heart beating faster than usual?”), followed by a confidence judgment expressed via a 10-points Likert scale (Figure 1). They were then asked to freely describe what they were doing in the half-hour prior to answering the question. The answers to the free text were coded according to the activity participants declared they were engaged in and were either labeled as high intensity (e.g., physical intensity, such as “workout” or “exercise,” as well as emotional intensity, such as “stressed,” “upset”). These labeled answers were then excluded from the analysis to avoid any confounding effect deriving from knowledge of one’s own heart rate. Participants were made aware that notifications would occur both when their heart-rate was elevated as well as when it was normal.

Notifications were sent to the notification center of the participant’s mobile at most once every 10 min (i.e., when the heart rate data was obtained from the wearable device). The probability of receiving a notification was dependent on a set of parameters: notifications sent each day to participants should not exceed six per day on average and they should be representative of the full range of heart rate values throughout the day. To this end, the probability was set to be inversely proportional to the empirical frequency of the current heart rate for that user, given the time of the day. Specifically, each day was split into segments of 4 h each and the possible values of heart rate were divided into buckets, each corresponding to a range of 5 beats per minute (BPM) each. Each new heart rate measurement was then assigned to the corresponding segment of the day and heart rate bucket. The empirical frequency of the new heart rate measurement within each segment and bucket was then computed as follows:

$$e\,m\,p\,i\,r\,i\,c\,a\,l\,f\,r\,e\,q\,u\,e\,n\,c\,y=\frac{h\,e\,a\,r\,t\,r\,a\,t\,e\,m\,e\,a\,s\,u\,r\,e\,m\,e\,n\,t\,s\,w\,i\,t\,h\,i\,n\,d\,a\,y\,s\,e\,g\,m\,e\,n\,t\,a\,n\,d\,b\,u\,c\,k\,e\,t}{h\,e\,a\,r\,t\,r\,a\,t\,e\,m\,e\,a\,s\,u\,r\,e\,m\,e\,n\,t\,s\,w\,i\,t\,h\,i\,n\,d\,a\,y\,s\,e\,g\,m\,e\,n\,t}$$

The probability of sending a notification to the participant was then calculated as being inversely proportional to the empirical frequency (if the resulting value was greater than one, the notification was sent with probability equal to one). To obtain an average of approximately six notifications per day, the constant of proportionality was fixed to a base probability of 20% (a value obtained heuristically) multiplied by the ratio of the number of desired notifications (6) and the number of heart rate measurements per day (72, given by six measurements per hour multiplied by the 12 h included in the 9 am to 9 pm range). Therefore, the final probability of sending a notification to a participant was computed as:

$$P=20\%\times \frac{6}{6\times 12}\times \frac{1}{e\,m\,p\,i\,r\,i\,c\,a\,l\,f\,r\,e\,q\,u\,e\,n\,c\,y}=1.67\%\times \frac{h\,e\,a\,r\,t\,r\,a\,t\,e\,m\,e\,a\,s\,u\,r\,e\,m\,e\,n\,t\,s\,w\,i\,t\,h\,i\,n\,d\,a\,y\,s\,e\,g\,m\,e\,n\,t}{h\,e\,a\,r\,t\,r\,a\,t\,e\,m\,e\,a\,s\,u\,r\,e\,m\,e\,n\,t\,s\,w\,i\,t\,h\,i\,n\,d\,a\,y\,s\,e\,g\,m\,e\,n\,t\,a\,n\,d\,b\,u\,c\,k\,e\,t}$$

To compute sensible probabilities at the beginning of the experiment, each participant started with heart rate measurements set to an *a priori* value. Specifically, in each segment of the day, the count was initialized to 1 measurement per bucket, for all buckets from 40 BPM to 90 BPM, and 0 measurements for all other buckets. These values were then updated with each new empirical measurement.

At the end of the study, participants were asked to answer a set of questions aimed at assessing participants’ strategy when replying to the questions throughout the study (see section “End of Study Questions” in Supplementary Material).

### Data Analysis

Responses to the heart rate judgment were analyzed separately for each participant to obtain a summary statistic using the Mann–Whitney *U* test. Specifically, given a response (A) where a participant reports perceiving their heart rate as higher than usual and a second response (B) where the same participant reports their heart rate as normal, if the recorded heart rate associated with the answer A is higher than the recorded heart rate associated with response B, then A and B formed a concordant pair. In the opposite case, i.e., where the recorded heart rate associated with A is lower than the one associated with B, these two responses constituted a discordant pair. The Mann–Whitney *U* test does not require any particular assumption about the probability distribution of heart rate values and participants’ answers, as long as they are independent under the null hypothesis and higher heart rate values increase the probability of positive answers under the alternative hypothesis. This is especially relevant since heart rate values were not sampled uniformly: the sampling depended both on the notifications schedule as well as on the number of completed trials. The test used the proportion of concordant versus discordant pairs (effect size), and compared it against the distribution of reference (according to the null hypothesis in which answers were independent of heart rate, and given the total number of pairs), generating a *p*-value for each participant. If such value was lower than 0.05 (a threshold yielding 5% false positive rate) that participant was deemed interoceptive. Effect sizes were calculated as the difference between concordant and discordant pairs, divided by the total number of pairs, and constituted participants’ interoceptive scores. Empirical cumulative distributions were calculated and plotted to illustrate the results.

As for confidence judgments, a threshold approach was implemented given no threshold-free method was identified. Participants’ average heart rate throughout the study was obtained. Data points higher than the computed standard deviation from such average for each participant were considered higher than usual (and normal otherwise). Hits, false alarms, correct rejections, and misses were calculated by comparing whether participants’ judgments (i.e., the yes or no response to the question “Is your heart beating faster than usual?”) were in line with measured heart rate (i.e., higher than usual or normal; see Table 1).

**TABLE 1:** Summary of type 1 responses based on participants’ answers to the question “Is your heart beating faster than usual?”(yes/no answer) and whether their heart rate was detected as higher than usual.

	Yes	No
Higher heart rate	Hit	Miss
Normal heart rate	False alarm	Correct rejection

The response type (i.e., correct when participants’ answer was a hit or a correct rejection, incorrect otherwise) and the confidence judgments (as a predictor) were used as arguments for the ROC function (pROC package for R) and area under the curve (AUC) values were extracted. A one-sample *t*-test was then conducted to check whether these AUROC values significantly differed from 0.5 (i.e., chance). Empirical cumulative distributions were calculated and plotted to illustrate the results.

To explore the relationship between subjective and objective interoceptive accuracy, a Pearson correlation between IAS scores and the effect sizes obtained from the Mann–Whitney *U* test described above was carried out. Furthermore, a correlation between such values and BPQ scores was conducted in order to investigate the relationship between subjective and objective interoceptive attention.

Finally, exploratory correlational analyses with Pearson correlations, aimed at identifying potential relationship between interoceptive abilities and mental health (i.e., DASS-21 and WEMWBS), as well as investigating the assumed relationship between interoception and BMI, were carried out.

Data was analyzed using Python and R, and plotted using base R.

## Results

Out of 1576 total trials, 181 trials, associated with high intensity activities, were excluded from the analyses. The minimum number of trials per participant in the overall sample was eight and the maximum 149 (*M* = 58.57, *SD* = 37.99), with only three participants answering to fewer than 15 trials.

**FIGURE 2:**
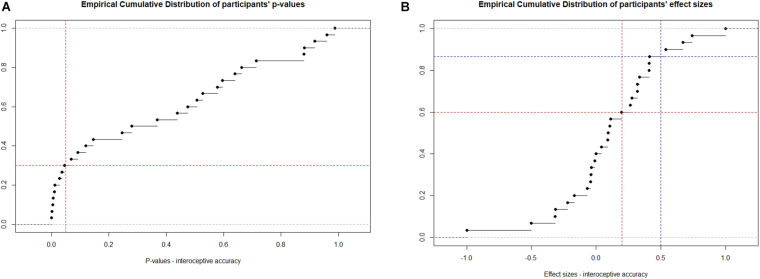
Empirical cumulative distribution (ECD) of **(A)** *p*-values obtained after comparing the Mann–Whitney *U* statistics against the population of reference and **(B)** effect sizes. **(A)** Red line vertical = 0.05, horizontal = 0.05 of the ECD; **(B)** Red line vertical = 0.2 (small effect size), horizontal = 0.2 of the ECD; Blue line vertical = 0.5 (medium effect size), horizontal = 0.5 of the ECD. Gray lines: upper and lower boundaries of *p*-values.

In terms of interoceptive accuracy, 1/3 of the sample (9/30 participants) obtained a *p*-value below the 0.05 threshold (Figure 2A). This result is in line with previous findings showing that approximately 1/3 of healthy participants is classified as interoceptive in interoceptive accuracy tasks (Brener and Ring, 2016). Given that participants had varying amounts of trials, the *p*-value (which takes into consideration the number of trials) was used to determine whether participants were interoceptive or not rather than relying on effect sizes. A small effect size was detected in slightly more than 1/3 of the sample and a medium effect size in 4/30 participants (Figure 2B).

**FIGURE 3:**
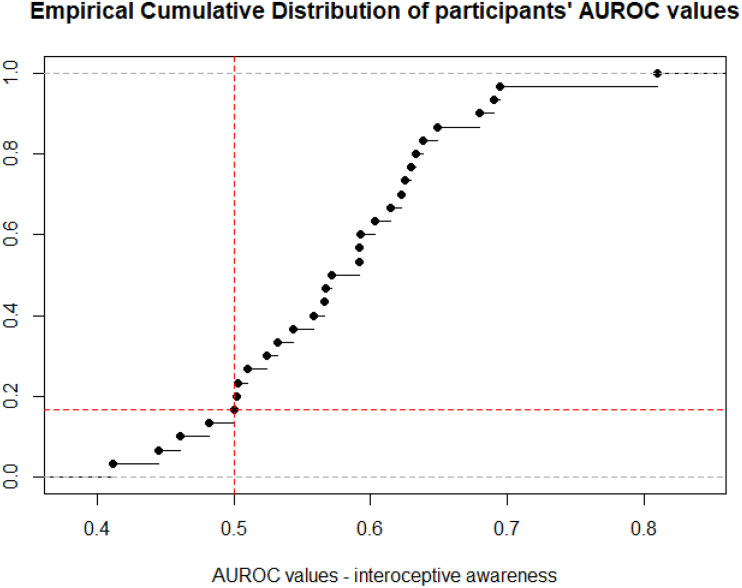
Empirical cumulative distribution of participants AUROC values obtained by applying the ROC function to correct and incorrect responses predicted by confidence ratings in such answers. Red lines: vertical = 0.5, horizontal = 0.5 of the ECD. Gray lines: upper and lower boundaries of effect sizes.

A one sample *t*-test revealed that participants’ interoceptive awareness, indexed via AUROC values, was statistically different from chance (*t*_29_ = 4.96, *p* < 0.001, *d* = 5.28; *M* = 0.58, *SD* = 0.09). As can be observed in Figure 3, in 4/30 participants, higher confidence ratings signaled decreased likelihood of correctly judging their heart rate as being higher than usual. As for the rest of the sample, their AUROC values suggested that higher confidence ratings were able to predict correct responses, in line with the notion that participants have insight into their interoceptive processes.

None of the correlations between interoceptive accuracy (effect sizes) and subjective interoception (IAS and BPQ), BMI as well as self-reported measures of mental health was significant (see Supplementary Table 1, also including descriptive statistics of all measures).

In terms of the qualitative checks, 5/30 participants reported having tried to manually check their pulse throughout the study. Specifically, one participant reported having checked their pulse the majority of the times (90%), whereas the others reported percentages between 10 and 20. Only one of these participants, who reported manually checking their pulse 10% of the times (out of 27 valid trials), was classified as interoceptive. When asked to provide a definition of usual heart rate (as compared to higher), all the participants reported that they considered “usual” as coinciding with their resting heart rate (i.e., their heart rate when sitting or when not engaged in intense activities).

## Discussion

The current study presented the development and proof of concept testing of an ecological test of interoceptive abilities, the CARED task. Approximately 1/3 of the participants, in line with the literature (Brener and Ring, 2016), were classified as interoceptive. In terms of interoceptive awareness, the majority of the sample showed insights into their performance, with higher confidence predicting increased likelihood of correct responses. Exploratory analyses aimed at identifying relationships between objective and subjective components of interoception, as well as self-reported mental health components, did not yield significant results.

We classified participants as interoceptive following a threshold-based approach due to the novelty of the task and the varying number of trials participants completed. Accordingly, the comparison of the obtained Mann–Whitney *U* statistic to the distribution of reference is dependent on the number of trials, whereby fewer trials make it more difficult for participants to be classified as interoceptive (correctly or not). However, instead of an arbitrary threshold, methods such as Gaussian mixture models or clustering algorithms may be preferred in future studies with a bigger sample size, allowing for a data-driven classification of participants between interoceptive and non-interoceptive.

By sampling cardiac judgments over time and “day-to-day” fluctuations in the cardiovascular system, this novel task captures interoceptive ability across an ecological range of afferent cardiac signals. This is a strength over existing procedures, such as the HCT and HDT, which could only index interoceptive accuracy at particular states of the cardiovascular system (e.g., at complete rest or after exercise). Despite this, research on the role of interoception in psychopathology has largely relied on the HCT and HDT. Therefore, a priority for future research is to assess whether existing associations between interoception and emotional constructs still hold when interoception is assessed in ecological conditions.

In order to exclude the potential confounding factor of inferring one’s own heart rate according to the activity one is involved in or the emotional state one is experiencing, we eliminated all answers associated with free text entries indicating high physical or emotional intensity in the 30 min preceding participants’ judgment. Furthermore, asking questions throughout the day and with an unpredictable pattern makes it difficult for participants to make their judgments solely based on estimation. Even though these countermeasures ensured adequate control for obvious activities and emotional states, it is not possible to completely exclude the possibility that participants’ judgment was not entirely based on perception but also on inference of one’s own states. However, the trade-off between measurement accuracy and the ecological nature of the task makes the CARED a valid method to continuously collect interoceptive data with minimal input from participants. The obvious advantage of such a simple methodology is the ease of use in clinical populations as well as in developmental studies.

In terms of interoceptive awareness, out of 30 participants, four presented with a profile indicating an inverse relationship between confidence and accuracy. The rest of the sample, however, behaved in line with the expectation that higher confidence is associated with correct responses (and vice-versa). In the current study, due to the low sample size, it wasn’t possible to investigate differences in interoceptive awareness in interoceptive versus non-interoceptive participants (e.g., Garfinkel et al., 2015). Future research is needed to elucidate the relationship between interoceptive performance and insight.

The lack of correlation between objective and subjective components of interoception corroborates the idea that these components dissociate and tap into different aspects of interoception (Murphy et al., 2019, 2020). The CARED task taps into both accuracy, as well as attention, components of interoception due to its ecological nature, thus making it more difficult to fully explore the relationship between the four components of the model. Future studies could focus on better distinguishing between objective accuracy and attention, perhaps by pairing the CARED with a task capturing time spent focusing on interoceptive sensations (e.g., experience sampling methods; Murphy et al., 2020). It has to be noted, however, that these findings may be due to the small sample size of the current study and hence further research is needed.

We did not find any correlation between interoceptive accuracy and self-reported measures of subjective wellbeing nor mental health dimensions. Whilst there is a significant amount of evidence that interoceptive disruption lies at the heart of several mental and physical health disorders (Dunn et al., 2010; Caspi et al., 2014; Quattrocki and Friston, 2014; Barrett and Simmons, 2015; Murphy et al., 2017; Khalsa et al., 2018), further research is needed to shed light on the exact relationship between interoceptive accuracy and subclinical manifestations. Furthermore, in the current study, participants were not screened for mental health disturbances prior to taking part in the study. Whilst this ensured a sample of healthy participants, it led to smaller between-subjects variations in mental health profiles. Future studies could employ stratification techniques to ensure subgroups of participants in different mental health categories. Furthermore, data-driven classifications between interoceptive and non-interoceptive participants could shed more light into differences in self-reported mental health subgroups.

When we looked at differences in heart rate judgments between participants who reported checking their pulse throughout the study and participants who did not, we found that only one of the interoceptive participants declared having checked their pulse a minimal portion of the time (roughly 10%). Nevertheless, manually checking one’s own pulse may not necessarily be related to one’s ability to detect changes in heart rate throughout the day and further research is needed to assess the impact of such strategies on interoceptive accuracy.

One direction for future research using the CARED task is to investigate whether prior familiarity with knowledge of one’s heart rate may improve task accuracy, speculatively by providing a more precise reference against which to compare heart rates during each trial. Secondly, some studies have reported that interoceptive ability can be trained, observing gains in accuracy following a heartbeat detection task with feedback (Garfinkel et al., 2017; Sugawara et al., 2020). Whether the CARED task accuracy can improve with either prior experience with one’s heart rate, or frequent monitoring of heart rate over several weeks or months, remains to be empirically observed.

In conclusion, we presented preliminary evidence that our novel task, the CARdiac Elevation Detection task, is able to measure interoceptive abilities in an ecological fashion and represents a potential asset for psychiatric and developmental research due to its ease of use and accessibility.

## Data Availability Statement

The original contributions presented in the study are included in the article/Supplementary Material, further inquiries can be directed to the corresponding author.

## Ethics Statement

The studies involving human participants were reviewed and approved by the Central University Research Ethics Committee, University of Oxford. The participants provided their written informed consent to participate in this study.

## Author Contributions

SP: conception and design of the study, analysis and interpretation of the data, and manuscript drafting and revisions. DM: conception and design of the study, analysis and interpretation of the data, and write-up revisions. CS: acquisition and interpretation of the data and write-up revisions. MC: design of the study, analysis and interpretation of the data, and write-up revisions. DP: conception and design of the study and write-up revisions. All authors approved the submitted version of the manuscript.

## Conflict of Interest

SP, DM, MC, and DP are employees of Huma Therapeutics Ltd. The remaining author declares that the research was conducted in the absence of any commercial or financial relationships that could be construed as a potential conflict of interest.

## Publisher’s Note

All claims expressed in this article are solely those of the authors and do not necessarily represent those of their affiliated organizations, or those of the publisher, the editors and the reviewers. Any product that may be evaluated in this article, or claim that may be made by its manufacturer, is not guaranteed or endorsed by the publisher.
