# Supplementary material for: Measuring Interoception: The CARdiac Elevation Detection Task
Source: Front Psychol. 2021 Aug 19;12:712896. doi: 10.3389/fpsyg.2021.712896 (PMC8416769; doi:10.3389/fpsyg.2021.712896)
Supplement: Supplementary file 1 [file Table_1.docx]

Supplementary Materials

1. Methods

1.1. End of study questions

At the end of the procedure, participants were asked the following questions:

1. To answer the questions during the study, did you ever manually check your pulse by touching your fingers against your wrist or neck? If yes, how often did you do this? Please estimate from 0% to 100%, how many of the questions you answered by manually checking your pulse.
2. Did you use any other methods to find out your heart rate?
3. The questions in this study asked you “Is your heart beating faster than usual?”. Different people can have different ideas of what “usual” heart rate is. For example, you could say that your heart rate when resting is your "usual". Or, "usual" might be what you feel your heart rate normally is at this time of the day.
    Could you please briefly explain what you thought “usual” heart rate meant when answering the questions?

2. Results

Table S1. Behavioural and questionnaires descriptive statistics and correlations between interoceptive accuracy (effect sizes) and BMI and self-reported measures of interoception and mental health.

| Variable | M | *SD* | R | *p* |
| --- | --- | --- | --- | --- |
| BMI | 22.12 | 3.23 | -0.35 | 0.06 |
| BPQ | 68.77 | 18.11 | -0.29 | 0.12 |
| IAS | 81.63 | 9.95 | -0.18 | 0.33 |
| WEMWBS | 48.67 | 6.90 | 0.07 | 0.70 |
| DASS-21 - Stress | 6 | 3.43 | -0.33 | 0.07 |
| DASS-21 - Anxiety | 3.4 | 2.19 | -0.17 | 0.37 |
| DASS_21 - Depression | 4.3 | 3.72 | -0.30 | 0.10 |
| STAI | 12.47 | 3.17 | 0.27 | 0.15 |
| CARED p values | 0.37 | 0.12 | - | - |
| CARED effect sizes | 0.34 | 0.39 | - | - |
